# Effects of Factors Related to Shift Work on Depression and Anxiety in Nurses

**DOI:** 10.3389/fpubh.2022.926988

**Published:** 2022-07-11

**Authors:** Yuxin Li, Yongchao Wang, Xiaoyan Lv, Rong Li, Xiangyun Guan, Li Li, Junli Li, Yingjuan Cao

**Affiliations:** ^1^School of Nursing and Rehabilitation, Cheeloo College of Medicine, Shandong University, Jinan, China; ^2^Department of Biostatistics, School of Public Health, Cheeloo College of Medicine, Shandong University, Jinan, China; ^3^Institute for Medical Dataology, Shandong University, Jinan, China; ^4^Department of Nursing, Qilu Hospital, Cheeloo College of Medicine, Shandong University, Jinan, China; ^5^Nursing Theory and Practice Innovation Research Center, Cheeloo College of Medicine, Shandong University, Jinan, China

**Keywords:** China, nurses, shift work, anxiety, depression, cross-sectional study

## Abstract

**Background:**

Although shift work is the foundation of the provision of 24-h continuous care in hospitals, it can negatively impact mental health in hospital workers such as nurses. Despite the connection between mental health and overall health, little is known about the effect of shift work-related factors on mental health in this population.

**Objectives:**

We investigated the effect of scheduling practices, physical and psychological characteristics related to shift work, and personal habits during shift work on depression and anxiety among nurses.

**Methods:**

In this multi-center cross-sectional study, 11,061 nurses from 20 hospitals in the Shandong Province of China completed an online survey between December 2020 and February 2022. Multivariate ordered logistic regression analysis was performed to examine shift-related factors associated with depression and anxiety in the study population.

**Results:**

The completion rate of all nurses' questionnaires was 83.00% (*n* = 9,181). Among the 9,181 respondents, 66.20% (*n* = 6,078) were shift nurses. Depression and anxiety were found in 58.82 and 62.08% of shift nurses, respectively, and these rates were influenced by fatigue during shift work, psychological stress before/during/after night shifts, feeling of being refreshed after resting before/after night shifts, using sleep medication before/after night shifts, physical discomfort during night shifts, busyness during night shifts, food intake during shift work, working > 40 h/week during shift work, and sleep quality before/after night shifts.

**Conclusions:**

Depression and anxiety in shift nurses may be addressed by reducing their workload, sources of stress during night shifts, and facilitating rest and relaxation.

## Introduction

Shift workers alternate and rotate morning, afternoon, and night shifts, and often work outside standard h (i.e., 7:00 to 18:00) (1). Given the demand for medical services, most healthcare sectors rely on shift workers, including core staff such as nurses (2). Shift work may negatively impact mental and physical health (3–5) because it disrupts the sleep-wake cycle (circadian rhythm) (6, 7). Mental health, which is associated with overall health and wellbeing, is affected by demographic, biological, psychosocial, and genetic factors, and by circadian rhythms (8–10). Mental health disorders are a main cause of illness-related absenteeism or presenteeism worldwide (11, 12), and poor mental health has been more strongly linked with absenteeism than physical illness or injury (8, 13, 14). Therefore, understanding how shift work influences mental health is essential for improving the occupational health and working conditions of shift workers.

Although chronic health conditions have been increasingly related to shift work, conclusions linking shift work with mental health have been inconsistent (7). Driesen et al. found that poor mental health was positively correlated with shift work (8). For instance, symptoms of depression were two times more frequent in shift workers compared with those who worked regular daytime h (8). Rosenberg et al. also found that adverse mental health issues were more common in shift workers than in non-shift workers, and that shift work carried a 33% increase in the risk of depressive symptoms (15). Furthermore, these workers had an increased risk of anxiety (although this was not statistically significant) (15). Sweeney et al. found *via* subgroup analysis that in comparison with non-shift workers, female shift workers had a 21% greater chance of reporting signs of major depression (7). This difference was not significant among male shift workers (7). Although previous reports have indicated that shift workers have an increased risk of depression and anxiety (16, 17), Øyane et al. reported that working at night was not linked with heightened depression and anxiety (18). Surprisingly, some studies have found that mental health in shift workers was superior to that in workers who did not do shift work (19, 20). These contradictory results may be explained by differences in occupational, industrial, and shift work characteristics across countries. Healthcare workers have been exposed to highly stressful work environments as a consequence of the COVID-19 pandemic, and this is likely to have affected their mental health (11). Nurses, as the mainstay of the healthcare workforce, are constantly exposed to workplace stressors that can have negative psychological effects such as anger, anxiety, depression, burnout, and irritability (21). Therefore, we examined how shift work, one of workplace stressors, could have impact on mental health in nurses in the present study.

A previous meta-analysis of longitudinal studies included a sensitivity analysis for the effect of gender on mental health in nurses (22). However, the authors did not analyze the characteristics associated with shift work because this information was not available (22). To fully explore the mechanisms by which shift work might influence mental health, it is necessary to consider specific characteristics of the working environment (e.g., fixed/permanent shifts, number of night shifts worked, start times, and the speed and direction of shift rotation) (23). The data could enable us to develop appropriate interventions. Therefore, in addition to collecting general demographic data from nurses, we aimed to explore detailed characteristics of shift work in terms of mental health.

Given that several studies with large samples have recently associated mental health with shift work (7, 24, 25), we attempted to explore how shift work might be related to depression and anxiety among Chinese nurses using data from a large multi-center sample from the Nurses' Health Cohort Study of Shandong. Our primary objective was to evaluate and describe the mental health status of Chinese nurses, including symptoms of depression and anxiety, while focusing on the effects of shift work-related characteristics such as psychological stress during or associated with shift work, work schedules, and personal habits during shift work on mental health outcomes. Finally, we sought to provide a framework for the development and implementation of health promotion programs and policies in the workplace, as well as to provide recommendations regarding the reduction of mental health risks associated with shift work.

## Methods and Materials

### Setting, Design, and Participants

In this multi-center cross-sectional study, we used baseline data collected from the Nurses' Health Cohort Study of Shandong (registration number: ChiCTR2100043202) (26) between December 2020 to February 2022. Health data were collected from nurses in 20 hospitals and eight cities in Shandong Province. The study participants were registered qualified nurses who volunteered to participate. Participants were excluded if they were nurses who had (a) retired or were practice nurses, (b) had <6 months of work experience, or (c) were on work leave during the investigation.

### Measures

#### Demographic Characteristics of Nurses

We examined demographic characteristics of the study population including age, department, education, gender, marital status, monthly income, professional title (the title of a technical or professional post), shift work (yes/no), and years of working as a nurse.

#### Characteristics of Shift Work

We classified the characteristics of shift work into three main categories: shift scheduling, shift work-related physical and mental characteristics, and personal habits during shift work. Therefore, the variables collected in this study included work schedule (two shifts/three shifts), shift rotation direction (counterclockwise/clockwise), shift work experience, months in which shift work was undertaken per year, the number of night shifts each month, interval between night shifts, night shift staffing, psychological stress before/during/after night shifts (yes/no), feeling of being refreshed after resting before/after night shifts (yes/no), use of medication to aid sleep before/after night shifts (yes/no), physical discomfort during night shifts (yes/no), busyness during night shifts (yes/no), food intake during shift work (normal/more/less), meal timing during shift work (regular/early/delayed), working >40 h/week during shift work (0–4 weeks/month), sleep quality before/after night shifts (normal/poor), and resting during night shifts (yes/no).

#### Mental and Physical Fatigue in Nurses During Shift Work

The 14-item Fatigue Scale (FS-14) is widely used to measure the degree of fatigue. The FS-14 was developed by Chalder et al. (27), and the Chinese version was created by Wang et al. (28). Its Cronbach's α was 0.773 and the test-retest reliability was 0.745 (28), which indicated that the test was culturally sensitive and valid (29). The scale consists of 14 questions with “yes” (1 point) or “no” (0 point) answers that correspond to the two dimensions of mental and physical fatigue. A higher score on this scale reflects a greater fatigue level. The Cronbach's α of the FS-14 was 0.803 in this study.

#### Anxiety Severity

The Generalized Anxiety Disorder 7-Item Scale (GAD-7), developed by Spitzer et al., with a test-retest reliability of 0.830 and a Cronbach's α of 0.920 indicating good reliability and validity, has been widely employed to screen for the presence and severity of anxiety (30). The total GAD-7 score ranges from 0–21 points, and each of the seven items is scored from zero (not at all) to three (nearly every day) such that 0–4, 5–9, 10–14, and 15–21 points represent normal, mild, moderate, and severe anxiety, respectively (30). The reliability and validity of the Chinese version of the GAD-7 were verified by He et al., who found that the test-retest reliability was 0.856 and the Cronbach's α was 0.898 (31). The Cronbach's α of the GAD-7 was 0.937 in this study.

#### Depression Severity

The 9-item Patient Health Questionnaire (PHQ-9) has been widely employed to measure depression symptoms (32). Kroenke et al. tested the reliability and validity of the PHQ-9 in medical institutions, and found the test-retest reliability to be 0.840 and the Cronbach's α of the questionnaire to be 0.890 (33). The total PHQ-9 score ranges from 0–27 points, and each of the 9 items is scored from zero (not at all) to three (nearly every day) such that 0–4, 5–9, 10–14, 15–19, and 20–27 points represent normal, mild, moderate, moderately severe, and severe depression, respectively (33). The reliability and validity of the Chinese version of the PHQ-9 for use with the general population were verified by Wang et al., who found a test-retest reliability of 0.860 and a Cronbach's α of 0.860 (34). The Cronbach's α of the PHQ-9 was 0.923 in this study.

### Data Collection

With the support of the Health Commission of Shandong Province, the research team signed a research project cooperation agreement with the participating hospitals and obtained the informed consent of each participant. In this web-based questionnaire survey, the participants first registered their personal information using the official WeChat account of the Nurses' Health Cohort Study of Shandong. They then completed the electronic questionnaire sent by the WeChat official account. The questionnaire took about 10 to 15 min to complete. During data collection, the research team set up an independent cloud server and a MySQL database cluster. The collected electronic questionnaire data were stored in the database in real time using security encryption technology, and the data were protected *via* a disaster recovery backup mechanism. Data managers could access the data platform of Nurses' Health Cohort Study *via* an authentication mechanism to perform data interface calls, data maintenance, and data status monitoring.

### Ethical Considerations

Participation in this study was voluntary, and the nurses provided electronic informed consent. The Shandong University Qilu Hospital Medical Ethics Committee approved the study (Registration number KYLL-202011-085).

### Data Analysis

We conducted statistical analyses using R software version 4.0.5 (R Development Core Team, Vienna, Austria). This study had two dependent variables including depression and anxiety. The analysis had the following steps: First, the effect of shift work on depression and anxiety was verified in all nurses. Second, we explored the effects of different characteristics of shift work on depression and anxiety in shift nurses.

We used descriptive statistics to assess continuous variables [calculated as “mean ± standard deviation (SD)”] and categorical variables (calculated as “frequencies” and “percentages”). We performed one-way analysis of variance (ANOVA) to compare depression and anxiety levels in nurses according to different continuous variables. We assessed the distributions of characteristics between the different groups using the chi-square test for categorical variables. The Bonferroni correction was applied for correcting the multiple tests. Therefore, *p*-value <0.006 was considered statistically significant for the univariate analysis of demographic characteristics related to depression/anxiety in all nurses, and *p*-value <0.002 was considered statistically significant for the univariate analysis of demographic and shift work characteristics related to depression/anxiety in shift nurses.

We assessed the existence of multicollinearity by calculating the variance inflation factor (VIF) for all variables, and then performed multivariable ordered logistic regression analysis (35). If the univariate analysis revealed statistically significant variables, we decided whether to enter them into a multivariable ordinal logistic regression analysis. The goal of this analysis was to identify the main variables associated with depression and anxiety and to estimate the adjusted odds ratios (ORs) and 95% confidence intervals (CIs). According to Bonferroni correction, *p-*value <0.025 was considered statistically significant for the regression analysis.

## Results

### Prevalence and Characteristics of Depression and Anxiety in Nurses

Our analysis included data from a total of 11,061 nurses from 20 hospitals in Shandong Province, China. Questionnaires with incomplete or inconsistent responses were excluded from the analysis, resulting in a completion rate of 83.00% (*n* = 9,181). See Tables 1, 2 for summaries of the descriptive statistics related to the prevalence of depression and anxiety and the basic demographic characteristics of the nurses. Data from 9,181 participants were analyzed. Approximately 94.76% (*n* = 8,700) of the participants were women, 57.10% (*n* = 5,242) held primary titles, the average age was 33.24 ± 7.31 years, and the average length of service was 10.75 ± 7.61 years. The results showed that 55.85% (*n* = 5,128) of the nurses had some degree of anxiety (mild = 41.19%, moderate = 9.93%, severe = 4.73%), and 58.39% (*n* = 5,361) had some degree of depression (mild = 39.61%, moderate = 8.80%, moderately severe = 6.61%, severe = 3.37%).

**Table 1:** Descriptive statistics and univariate analysis of demographic characteristics related to depression in all nurses (*N* = 9,181).

**Variables**	**Total, *n* (%)**	**Depression, *n* (%)**					***F*/χ^2‡^**	***p* value**
		**Normal**	**Mild**	**Moderate**	**Moderately severe**	**Severe**		
Total	9,181 (100.00)	3,820 (41.61)	3,637 (39.61)	808 (8.80)	607 (6.61)	309 (3.37)		
Age, years (mean ± SD)	33.24 ± 7.31	33.58 ± 7.93	33.08 ± 6.81	33.20 ± 7.18	32.44 ± 6.49	32.58 ± 6.61	4.92	**<0.001**
Years of working (mean ± SD)	10.75 ± 7.61	11.19 ± 8.23	10.48 ± 7.16	10.67 ± 7.41	9.96 ± 6.77	10.21 ± 6.61	6.30	**<0.001**
**Gender**								
Male	481 (5.24)	174 (36.17)	178 (37.01)	53 (11.02)	53 (11.02)	23 (4.78)	25.25	**<0.001**
Female	8,700 (94.76)	3,646 (41.91)	3,459 (39.76)	755 (8.68)	554 (6.37)	286 (3.29)		
**Education**								
Associate's degree	922 (10.04)	420 (45.55)	334 (36.23)	78 (8.46)	64 (6.94)	26 (2.82)	8.58	0.379
Bachelor's degree	8,149 (88.76)	3,356 (41.18)	3,260 (40)	720 (8.84)	535 (6.57)	278 (3.41)		
Master's degree	110 (1.20)	44 (40)	43 (39.09)	10 (9.09)	8 (7.27)	5 (4.55)		
**Marital status**								
Unmarried	2,132 (23.22)	870 (40.81)	844 (39.59)	194 (9.1)	153 (7.18)	71 (3.33)	4.23	0.836
Married	6,861 (74.73)	2,872 (41.86)	2,719 (39.63)	598 (8.72)	438 (6.38)	234 (3.41)		
Others	188 (2.05)	78 (41.49)	74 (39.36)	16 (8.51)	16 (8.51)	4 (2.13)		
**Department**								
Internal medicine	2,678 (29.17)	1,063 (39.69)	1,083 (40.44)	241 (9)	182 (6.8)	109 (4.07)	73.49	**<0.001**
Surgery	1,998 (21.76)	820 (41.04)	786 (39.34)	172 (8.61)	147 (7.36)	73 (3.65)		
Emergency	498 (5.42)	193 (38.76)	187 (37.55)	62 (12.45)	39 (7.83)	17 (3.41)		
Gynecology and obstetrics	672 (7.32)	297 (44.2)	278 (41.37)	50 (7.44)	33 (4.91)	14 (2.08)		
Pediatrics	697 (7.59)	288 (41.32)	288 (41.32)	59 (8.46)	42 (6.03)	20 (2.87)		
Operating room	634 (6.91)	288 (45.43)	241 (38.01)	51 (8.04)	36 (5.68)	18 (2.84)		
Intensive care unit	573 (6.24)	200 (34.9)	240 (41.88)	56 (9.77)	53 (9.25)	24 (4.19)		
Outpatient	328 (3.57)	149 (45.43)	129 (39.33)	28 (8.54)	19 (5.79)	3 (0.91)		
Others	1,103 (12.01)	522 (47.33)	405 (36.72)	89 (8.07)	56 (5.08)	31 (2.81)		
**Professional title** ^†^								
Primary	5,242 (57.10)	2,195 (41.87)	2,058 (39.26)	441 (8.41)	355 (6.77)	193 (3.68)	59.27	**<0.001**
Intermediate	3,471 (37.81)	1,366 (39.35)	1,424 (41.03)	328 (9.45)	241 (6.94)	112 (3.23)		
Senior	468 (5.10)	259 (55.34)	155 (33.12)	39 (8.33)	11 (2.35)	4 (0.85)		
**Monthly income, CNY**								
<3,000	841 (9.16)	362 (43.04)	335 (39.83)	65 (7.73)	52 (6.18)	27 (3.21)	15.25	0.228
3,000–5,999	5,190 (56.53)	2,124 (40.92)	2,067 (39.83)	450 (8.67)	365 (7.03)	184 (3.55)		
6,000–8,999	2,473 (26.94)	1,023 (41.37)	975 (39.43)	236 (9.54)	160 (6.47)	79 (3.19)		
≥9,000	677 (7.37)	311 (45.94)	260 (38.4)	57 (8.42)	30 (4.43)	19 (2.81)		
**Shift work**								
No	3,103 (33.80)	1,515 (48.82)	1,148 (37.00)	224 (7.22)	147 (4.74)	69 (2.22)	123.16	**<0.001**
Yes	6,078 (66.20)	2,305 (37.92)	2,489 (40.95)	584 (9.61)	460 (7.57)	240 (3.95)		

*SD, standard deviation; CNY, China Yuan. Statistically significant differences after application of the Bonferroni correction are shown as bold numbers (p < 0.006)*.

† *Nursing professional titles can be divided into three categories: primary, intermediate and senior*.

‡ *F for one-way analysis of variance (ANOVA) and χ2 for chi-square test*.

**Table 2:** Descriptive statistics and univariate analysis of demographic characteristics related to anxiety in all nurses (*N* = 9,181).

**Variables**	**Total, *n* (%)**	**Anxiety, *n* (%)**				***F*/χ^2‡^**	***p* value**
		**Normal**	**Mild**	**Moderate**	**Severe**		
Total	9,181 (100.00)	4,053 (44.15)	3,782 (41.19)	912 (9.93)	434 (4.73)		
Age, years (mean ± SD)	33.24 ± 7.31	33.48 ± 7.79	33.14 ± 6.95	32.69 ± 6.83	32.98 ± 6.73	3.51	0.015
Years of working (mean ± SD)	10.75 ± 7.61	11.08 ± 8.12	10.56 ± 7.25	10.16 ± 7.00	10.49 ± 6.88	5.30	**0.001**
**Gender**							
Male	481 (5.24)	198 (41.16)	182 (37.84)	70 (14.55)	31 (6.44)	16.48	**<0.001**
Female	8,700 (94.76)	3,855 (44.31)	3,600 (41.38)	842 (9.68)	403 (4.63)		
**Education**							
Associate's degree	922 (10.04)	443 (48.05)	352 (38.18)	89 (9.65)	38 (4.12)	9.32	0.157
Bachelor's degree	8,149 (88.76)	3,569 (43.80)	3,378 (41.45)	810 (9.94)	392 (4.81)		
Master's degree	110 (1.20)	41 (37.27)	52 (47.27)	13 (11.82)	4 (3.64)		
**Marital status**							
Unmarried	2,132 (23.22)	935 (43.86)	880 (41.28)	236 (11.07)	81 (3.80)	9.38	0.153
Married	6,861 (74.73)	3,030 (44.16)	2,827 (41.20)	659 (9.61)	345 (5.03)		
Others	188 (2.05)	88 (46.81)	75 (39.89)	17 (9.04)	8 (4.26)		
**Department**							
Internal medicine	2,678 (29.17)	1,132 (42.27)	1,117 (41.71)	283 (10.57)	146 (5.45)	65.23	**<0.001**
Surgery	1,998 (21.76)	869 (43.49)	836 (41.84)	192 (9.61)	101 (5.06)		
Emergency	498 (5.42)	207 (41.57)	201 (40.36)	69 (13.86)	21 (4.22)		
Gynecology and obstetrics	672 (7.32)	321 (47.77)	275 (40.92)	46 (6.85)	30 (4.46)		
Pediatrics	697 (7.59)	296 (42.47)	309 (44.33)	64 (9.18)	28 (4.02)		
Operating room	634 (6.91)	291 (45.9)	261 (41.17)	57 (8.99)	25 (3.94)		
Intensive care unit	573 (6.24)	223 (38.92)	241 (42.06)	76 (13.26)	33 (5.76)		
Outpatient	328 (3.57)	171 (52.13)	126 (38.41)	23 (7.01)	8 (2.44)		
Others	1,103 (12.01)	543 (49.23)	416 (37.72)	102 (9.25)	42 (3.81)		
**Professional title** ^†^							
Primary	5,242 (57.10)	2,342 (44.68)	2,133 (40.69)	506 (9.65)	261 (4.98)	37.93	**<0.001**
Intermediate	3,471 (37.81)	1,455 (41.92)	1,475 (42.49)	379 (10.92)	162 (4.67)		
Senior	468 (5.10)	256 (54.7)	174 (37.18)	27 (5.77)	11 (2.35)		
**Monthly income, CNY**							
<3,000	841 (9.16)	388 (46.14)	331 (39.36)	89 (10.58)	33 (3.92)	5.37	0.801
3,000–5,999	5,190 (56.53)	2,274 (43.82)	2,145 (41.33)	515 (9.92)	256 (4.93)		
6,000–8,999	2,473 (26.94)	1,084 (43.83)	1,026 (41.49)	250 (10.11)	113 (4.57)		
≥9,000	677 (7.37)	307 (45.35)	280 (41.36)	58 (8.57)	32 (4.73)		
**Shift work**							
No	3,103 (33.80)	1,550 (49.95)	1,191 (38.38)	243 (7.83)	119 (3.83)	73.54	**<0.001**
Yes	6,078 (66.20)	2,503 (41.18)	2,591 (42.63)	669 (11.01)	315 (5.18)		

*SD, standard deviation; CNY, China Yuan. Statistically significant differences after application of the Bonferroni correction are shown as bold numbers (p < 0.006)*.

† *Nursing professional titles can be divided into three categories: primary, intermediate and senior*.

‡ *F for one-way analysis of variance (ANOVA) and χ2 for chi-square test*.

Among the 9,181 nurses, 66.20% (*n* = 6,078) were shift nurses. As shown in Tables 1, 2, the prevalence of depression and anxiety in shift nurses was statistically significantly higher than that among non-shift nurses. Among the shift nurses, 58.82% (*n* = 3,575) had some degree of anxiety (mild = 42.63%, moderate = 11.01%, severe = 5.18%), and 62.08% (*n* = 3,773) had some degree of depression (mild = 40.95%, moderate = 9.61%, moderately severe = 7.57%, severe = 3.95%).

Tables 3, 4 show the descriptive statistics of the prevalence of depression and anxiety, the basic demographic characteristics, and the characteristics of shift work in shift nurses. The average age and the average length of service of shift nurses were 31.06 ± 5.31 years and 8.42 ± 5.13 years, respectively. Among the shift nurses, 93.37% (*n* = 5,675) were women, 67.14% (*n* = 4,081) held primary titles, 53.09% (*n* = 3,227) worked in two shifts, and 60.50% (*n* = 3,677) had a schedule in which shifts were rotated in a clockwise direction.

**Table 3:** Descriptive statistics and univariate analysis of demographic and shift work characteristics related to depression in shift nurses (*N* = 6,078).

**Variables**	**Total**	**Depression, *n* (%)**					***F*/χ^2§^**	***p*value**
		**Normal**	**Mild**	**Moderate**	**Moderately severe**	**Severe**		
Total	6,078 (100.00)	2,305 (37.92)	2,489 (40.95)	584 (9.61)	460 (7.57)	240 (3.95)		
Age, years (mean ± SD)	31.06 ± 5.31	30.73 ± 5.51	31.18 ± 5.06	31.17 ± 5.27	31.53 ± 5.37	31.77 ± 5.63	4.61	**0.001**
Years of working (mean ± SD)	8.42 ± 5.13	8.24 ± 5.28	8.41 ± 4.93	8.54 ± 4.88	8.81 ± 5.35	9.24 ± 5.66	2.97	0.018
**Fatigue scores** **(mean ±SD)**								
Physical fatigue scores	5.48 ± 2.73	4.05 ± 2.84	6.14 ± 2.26	6.74 ± 1.80	6.76 ± 2.36	6.73 ± 2.52	320.92	**<0.001**
Mental fatigue scores	2.75 ± 1.44	2.18 ± 1.37	2.87 ± 1.36	3.52 ± 1.42	3.46 ± 1.17	3.67 ± 1.22	215.08	**<0.001**
**Gender**								
Male	403 (6.63)	136 (33.75)	151 (37.47)	48 (11.91)	47 (11.66)	21 (5.21)	16.95	0.002
Female	5,675 (93.37)	2,169 (38.22)	2,338 (41.2)	536 (9.44)	413 (7.28)	219 (3.86)		
**Education**								
Associate's degree	649 (10.68)	275 (42.37)	245 (37.75)	59 (9.09)	52 (8.01)	18 (2.77)	9.90	0.272
Bachelor's degree	5,392 (88.71)	2,019 (37.44)	2,228 (41.32)	521 (9.66)	404 (7.49)	220 (4.08)		
Master's degree	37 (0.61)	11 (29.73)	16 (43.24)	4 (10.81)	4 (10.81)	2 (5.41)		
**Marital status**								
Unmarried	1,811 (29.80)	710 (39.2)	733 (40.47)	173 (9.55)	132 (7.29)	63 (3.48)	6.34	0.609
Married	4,167 (68.56)	1,550 (37.2)	1,718 (41.23)	403 (9.67)	321 (7.7)	175 (4.2)		
Others	100 (1.65)	45 (45)	38 (38)	8 (8)	7 (7)	2 (2)		
**Department**								
Internal medicine	1,851 (30.45)	690 (37.28)	768 (41.49)	175 (9.45)	139 (7.51)	79 (4.27)	33.45	0.397
Surgery	1,472 (24.22)	557 (37.84)	594 (40.35)	143 (9.71)	121 (8.22)	57 (3.87)		
Emergency	396 (6.52)	141 (35.61)	154 (38.89)	53 (13.38)	31 (7.83)	17 (4.29)		
Gynecology and obstetrics	476 (7.83)	198 (41.6)	203 (42.65)	36 (7.56)	26 (5.46)	13 (2.73)		
Pediatrics	497 (8.18)	192 (38.63)	215 (43.26)	44 (8.85)	32 (6.44)	14 (2.82)		
Operating room	359 (5.91)	142 (39.55)	144 (40.11)	39 (10.86)	20 (5.57)	14 (3.9)		
Intensive care unit	499 (8.21)	174 (34.87)	204 (40.88)	49 (9.82)	50 (10.02)	22 (4.41)		
Outpatient	32 (0.53)	11 (34.38)	12 (37.5)	4 (12.5)	4 (12.5)	1 (3.12)		
Others	496 (8.16)	200 (40.32)	195 (39.31)	41 (8.27)	37 (7.46)	23 (4.64)		
**Professional title** ^†^								
Primary	4,081 (67.14)	1,613 (39.52)	1,640 (40.19)	377 (9.24)	290 (7.11)	161 (3.95)	19.19	0.014
Intermediate	1,960 (32.25)	676 (34.49)	838 (42.76)	201 (10.26)	167 (8.52)	78 (3.98)		
Senior	37 (0.61)	16 (43.24)	11 (29.73)	6 (16.22)	3 (8.11)	1 (2.7)		
**Monthly income, CNY**								
<3,000	556 (9.15)	226 (40.65)	230 (41.37)	44 (7.91)	34 (6.12)	22 (3.96)	15.44	0.218
3,000–5,999	3,790 (62.36)	1,474 (38.89)	1,530 (40.37)	354 (9.34)	292 (7.7)	140 (3.69)		
6,000–8,999	1,466 (24.12)	515 (35.13)	615 (41.95)	156 (10.64)	115 (7.84)	65 (4.43)		
≥9,000	266 (4.38)	90 (33.83)	114 (42.86)	30 (11.28)	19 (7.14)	13 (4.89)		
**Work schedule** ^‡^								
Two shifts	3,227 (53.09)	1,196 (37.06)	1,363 (42.24)	316 (9.79)	239 (7.41)	113 (3.5)	24.47	0.002
Three shifts	2,649 (43.58)	1,044 (39.41)	1,054 (39.79)	238 (8.98)	196 (7.4)	117 (4.42)		
Others	202 (3.32)	65 (32.18)	72 (35.64)	30 (14.85)	25 (12.38)	10 (4.95)		
**Shift rotation direction**								
Clockwise	3,677 (60.50)	1,463 (39.79)	1,477 (40.17)	327 (8.89)	267 (7.26)	143 (3.89)	16.12	0.003
Counterclockwise	2,401 (39.50)	842 (35.07)	1,012 (42.15)	257 (10.7)	193 (8.04)	97 (4.04)		
**Shift work experience**								
0–5 years	1,946 (32.02)	801 (41.16)	779 (40.03)	165 (8.48)	139 (7.14)	62 (3.19)	24.62	0.077
6–10 years	2,218 (36.49)	821 (37.02)	916 (41.3)	223 (10.05)	168 (7.57)	90 (4.06)		
11–15 years	1,406 (23.13)	493 (35.06)	598 (42.53)	147 (10.46)	108 (7.68)	60 (4.27)		
16–20 years	384 (6.32)	145 (37.76)	150 (39.06)	36 (9.38)	34 (8.85)	19 (4.95)		
>20 years	124 (2.04)	45 (36.29)	46 (37.1)	13 (10.48)	11 (8.87)	9 (7.26)		
**Months of shift work per year**								
1–3 months	289 (4.75)	137 (47.4)	108 (37.37)	32 (11.07)	11 (3.81)	1 (0.35)	53.19	**<0.001**
10–12 months	4,757 (78.27)	1,742 (36.62)	1,949 (40.97)	456 (9.59)	392 (8.24)	218 (4.58)		
7–9 months	538 (8.85)	218 (40.52)	231 (42.94)	46 (8.55)	32 (5.95)	11 (2.04)		
4–6 months	494 (8.13)	208 (42.11)	201 (40.69)	50 (10.12)	25 (5.06)	10 (2.02)		
**Night shifts per month**								
≤ 4	1,267 (20.85)	561 (44.28)	486 (38.36)	106 (8.37)	67 (5.29)	47 (3.71)	52.03	**<0.001**
5–9	4,150 (68.28)	1,538 (37.06)	1,724 (41.54)	412 (9.93)	319 (7.69)	157 (3.78)		
≥10	661 (10.88)	206 (31.16)	279 (42.21)	66 (9.98)	74 (11.2)	36 (5.45)		
**Interval between night shifts**								
≤ 4 days	1,955 (32.17)	714 (36.52)	797 (40.77)	198 (10.13)	164 (8.39)	82 (4.19)	9.00	0.342
5–7 days	3,474 (57.16)	1,332 (38.34)	1,424 (40.99)	325 (9.36)	261 (7.51)	132 (3.8)		
≥8 days	649 (10.68)	259 (39.91)	268 (41.29)	61 (9.4)	35 (5.39)	26 (4.01)		
**Night shift staffing**								
1	1,932 (31.79)	737 (38.15)	783 (40.53)	187 (9.68)	140 (7.25)	85 (4.4)	7.00	0.858
2 (take turns)	2,212 (36.39)	860 (38.88)	910 (41.14)	203 (9.18)	162 (7.32)	77 (3.48)		
2 (take no turns)	838 (13.79)	306 (36.52)	353 (42.12)	82 (9.79)	66 (7.88)	31 (3.7)		
≥3	1,096 (18.03)	402 (36.68)	443 (40.42)	112 (10.22)	92 (8.39)	47 (4.29)		
**Psychological stress before night shifts**								
No	633 (10.41)	428 (67.61)	145 (22.91)	30 (4.74)	18 (2.84)	12 (1.9)	266.25	**<0.001**
Yes	5,445 (89.59)	1,877 (34.47)	2,344 (43.05)	554 (10.17)	442 (8.12)	228 (4.19)		
**Psychological stress during night shifts**								
No	698 (11.48)	474 (67.91)	162 (23.21)	33 (4.73)	19 (2.72)	10 (1.43)	304.22	**<0.001**
Yes	5,380 (88.52)	1,831 (34.03)	2,327 (43.25)	551 (10.24)	441 (8.2)	230 (4.28)		
**Psychological stress after night shifts**								
No	2,546 (41.89)	1,336 (52.47)	889 (34.92)	181 (7.11)	99 (3.89)	41 (1.61)	451.09	**<0.001**
Yes	3,532 (58.11)	969 (27.43)	1,600 (45.3)	403 (11.41)	361 (10.22)	199 (5.63)		
**Using sleep medication before night shifts**								
Never/rarely	4,729 (77.81)	2,011 (42.52)	1,911 (40.41)	429 (9.07)	261 (5.52)	117 (2.47)	410.19	**<0.001**
Sometimes	1,130 (18.59)	237 (20.97)	511 (45.22)	135 (11.95)	159 (14.07)	88 (7.79)		
Often	219 (3.60)	57 (26.03)	67 (30.59)	20 (9.13)	40 (18.26)	35 (15.98)		
**Using sleep medication after night shifts**								
Never/rarely	4,930 (81.11)	2,074 (42.07)	2,006 (40.69)	442 (8.97)	277 (5.62)	131 (2.66)	401.41	**<0.001**
Sometimes	946 (15.56)	184 (19.45)	425 (44.93)	115 (12.16)	140 (14.8)	82 (8.67)		
Often	202 (3.32)	47 (23.27)	58 (28.71)	27 (13.37)	43 (21.29)	27 (13.37)		
**Physical discomfort during night shifts**								
No	1,935 (31.84)	1,116 (57.67)	630 (32.56)	93 (4.81)	69 (3.57)	27 (1.4)	515.92	**<0.001**
Yes	4,143 (68.16)	1,189 (28.7)	1,859 (44.87)	491 (11.85)	391 (9.44)	213 (5.14)		
**Busyness during night shifts**								
No	1,739 (28.61)	836 (48.07)	645 (37.09)	133 (7.65)	85 (4.89)	40 (2.3)	124.67	**<0.001**
Yes	4,339 (71.39)	1,469 (33.86)	1,844 (42.5)	451 (10.39)	375 (8.64)	200 (4.61)		
**Feeling of being refreshed after resting before night shifts**								
No	1,657 (27.26)	373 (22.51)	682 (41.16)	230 (13.88)	214 (12.91)	158 (9.54)	452.10	**<0.001**
Yes	4,421 (72.74)	1,932 (43.7)	1,807 (40.87)	354 (8.01)	246 (5.56)	82 (1.85)		
**Feeling of being refreshed after resting after night shifts**								
No	767 (12.62)	150 (19.56)	306 (39.9)	92 (11.99)	116 (15.12)	103 (13.43)	349.52	**<0.001**
Yes	5,311 (87.38)	2,155 (40.58)	2,183 (41.1)	492 (9.26)	344 (6.48)	137 (2.58)		
**Sleep quality before night shifts**								
Normal	3,996 (65.75)	1,787 (44.72)	1,599 (40.02)	299 (7.48)	223 (5.58)	88 (2.2)	350.45	**<0.001**
Poor	2,082 (34.25)	518 (24.88)	890 (42.75)	285 (13.69)	237 (11.38)	152 (7.3)		
**Sleep quality after night shifts**								
Normal	4,700 (77.33)	1,978 (42.09)	1,914 (40.72)	405 (8.62)	295 (6.28)	108 (2.3)	304.90	**<0.001**
Poor	1,378 (22.67)	327 (23.73)	575 (41.73)	179 (12.99)	165 (11.97)	132 (9.58)		
**Food intake during shift work**								
Normal	2,085 (34.30)	1,003 (48.11)	779 (37.36)	154 (7.39)	102 (4.89)	47 (2.25)	177.79	**<0.001**
More	749 (12.32)	252 (33.64)	291 (38.85)	88 (11.75)	69 (9.21)	49 (6.54)		
Less	3,244 (53.37)	1,050 (32.37)	1,419 (43.74)	342 (10.54)	289 (8.91)	144 (4.44)		
**Meal timing during shift work**								
Regular	2,302 (37.87)	1,068 (46.39)	896 (38.92)	172 (7.47)	123 (5.34)	43 (1.87)	168.73	**<0.001**
Early	1,008 (16.58)	372 (36.9)	393 (38.99)	98 (9.72)	88 (8.73)	57 (5.65)		
Delayed	2,768 (45.54)	865 (31.25)	1,200 (43.35)	314 (11.34)	249 (9)	140 (5.06)		
**Working > 40 h/week during shift work**								
Never/rarely	1,148 (18.89)	631 (54.97)	375 (32.67)	75 (6.53)	38 (3.31)	29 (2.53)	273.59	**<0.001**
1 week/month	1,160 (19.09)	461 (39.74)	496 (42.76)	100 (8.62)	78 (6.72)	25 (2.16)		
2 weeks/month	1,069 (17.59)	368 (34.42)	479 (44.81)	104 (9.73)	90 (8.42)	28 (2.62)		
3 weeks/month	638 (10.50)	219 (34.33)	267 (41.85)	70 (10.97)	62 (9.72)	20 (3.13)		
4 weeks/month	2,063 (33.94)	626 (30.34)	872 (42.27)	235 (11.39)	192 (9.31)	138 (6.69)		
**Rest during night shifts**								
No	1,868 (30.73)	702 (37.58)	714 (38.22)	169 (9.05)	175 (9.37)	108 (5.78)	40.36	**<0.001**
Yes	4,210 (69.27)	1,603 (38.08)	1,775 (42.16)	415 (9.86)	285 (6.77)	132 (3.14)		

*SD, standard deviation; CNY, China Yuan. Statistically significant differences after application of the Bonferroni correction are shown as bold numbers (p < 0.002)*.

† *Nursing professional titles can be divided into three categories: primary, intermediate and senior*.

‡ *Other work schedules include long-term irregular shifts for a variety of reasons*.

§ *F for one-way analysis of variance (ANOVA) and χ2 for chi-square test*.

**Table 4:** Descriptive statistics and univariate analysis of demographic and shift work characteristics related to anxiety in shift nurses (*N* = 6,078).

**Variables**	**Total**	**Anxiety, *n* (%)**				***F*/χ^2§^**	***p* value**
		**Normal**	**Mild**	**Moderate**	**Severe**		
Total	6,078 (100.00)	2,503 (41.18)	2,591 (42.63)	669 (11.01)	315 (5.18)		
Age, years (mean ± SD)	31.06 ± 5.31	30.79 ± 5.43	31.17 ± 5.14	31.26 ± 5.43	31.93 ± 5.37	5.74	**<0.001**
Years of working (mean ± SD)	8.42 ± 5.13	8.25 ± 5.20	8.46 ± 4.97	8.52 ± 5.33	9.31 ± 5.30	4.20	0.006
**Fatigue scores** **(mean ±SD)**							
Physical fatigue scores	5.48 ± 2.73	4.21 ± 2.85	6.23 ± 2.22	6.68 ± 2.26	6.75 ± 2.34	370.12	**<0.001**
Mental fatigue scores	2.75 ± 1.44	2.26 ± 1.40	2.95 ± 1.38	3.39 ± 1.28	3.54 ± 1.29	205.50	**<0.001**
**Gender**							
Male	403 (6.63)	162 (40.20)	150 (37.22)	65 (16.13)	26 (6.45)	14.69	0.002
Female	5,675 (93.37)	2,341 (41.25)	2,441 (43.01)	604 (10.64)	289 (5.09)		
**Education**							
Associate's degree	649 (10.68)	294 (45.3)	262 (40.37)	69 (10.63)	24 (3.70)	10.32	0.112
Bachelor's degree	5,392 (88.71)	2,198 (40.76)	2,308 (42.80)	596 (11.05)	290 (5.38)		
Master's degree	37 (0.61)	11 (29.73)	21 (56.76)	4 (10.81)	1 (2.70)		
**Marital status**							
Unmarried	1,811 (29.80)	772 (42.63)	766 (42.30)	206 (11.37)	67 (3.70)	17.14	0.009
Married	4,167 (68.56)	1,683 (40.39)	1,785 (42.84)	453 (10.87)	246 (5.90)		
Others	100 (1.65)	48 (48.00)	40 (40.00)	10 (10.00)	2 (2.00)		
**Department**							
Internal medicine	1,851 (30.45)	749 (40.46)	789 (42.63)	205 (11.08)	108 (5.83)	29.10	0.216
Surgery	1,472 (24.22)	604 (41.03)	638 (43.34)	151 (10.26)	79 (5.37)		
Emergency	396 (6.52)	156 (39.39)	168 (42.42)	55 (13.89)	17 (4.29)		
Gynecology and obstetrics	476 (7.83)	216 (45.38)	200 (42.02)	36 (7.56)	24 (5.04)		
Pediatrics	497 (8.18)	200 (40.24)	228 (45.88)	53 (10.66)	16 (3.22)		
Operating room	359 (5.91)	153 (42.62)	152 (42.34)	39 (10.86)	15 (4.18)		
Intensive care unit	499 (8.21)	195 (39.08)	205 (41.08)	70 (14.03)	29 (5.81)		
Outpatient	32 (0.53)	14 (43.75)	11 (34.38)	6 (18.75)	1 (3.12)		
Others	496 (8.16)	216 (43.55)	200 (40.32)	54 (10.89)	26 (5.24)		
**Professional title** ^†^							
Primary	4,081 (67.14)	1,742 (42.69)	1,703 (41.73)	427 (10.46)	209 (5.12)	14.57	0.024
Intermediate	1,960 (32.25)	744 (37.96)	875 (44.64)	238 (12.14)	103 (5.26)		
Senior	37 (0.61)	17 (45.95)	13 (35.14)	4 (10.81)	3 (8.11)		
**Monthly income, CNY**							
<3,000	556 (9.15)	243 (43.71)	229 (41.19)	63 (11.33)	21 (3.78)	16.06	0.066
3,000–5,999	3,790 (62.36)	1,588 (41.90)	1,598 (42.16)	410 (10.82)	194 (5.12)		
6,000–8,999	1,466 (24.12)	575 (39.22)	634 (43.25)	176 (12.01)	81 (5.53)		
≥9,000	266 (4.38)	97 (36.47)	130 (48.87)	20 (7.52)	19 (7.14)		
**Work schedule** ^‡^							
Two shifts	3,227 (53.09)	1,317 (40.81)	1,407 (43.6)	343 (10.63)	160 (4.96)	16.78	0.010
Three shifts	2,649 (43.58)	1,119 (42.24)	1,100 (41.53)	290 (10.95)	140 (5.29)		
Others	202 (3.32)	67 (33.17)	84 (41.58)	36 (17.82)	15 (7.43)		
**Shift rotation direction**							
Clockwise	3,677 (60.50)	1,572 (42.75)	1,517 (41.26)	392 (10.66)	196 (5.33)	11.10	0.011
Counterclockwise	2,401 (39.50)	931 (38.78)	1,074 (44.73)	277 (11.54)	119 (4.96)		
**Shift work experience**							
0–5 years	1,946 (32.02)	853 (43.83)	806 (41.42)	213 (10.95)	74 (3.80)	20.58	0.057
6–10 years	2,218 (36.49)	901 (40.62)	952 (42.92)	244 (11.00)	121 (5.46)		
11–15 years	1,406 (23.13)	547 (38.9)	615 (43.74)	158 (11.24)	86 (6.12)		
16–20 years	384 (6.32)	158 (41.15)	163 (42.45)	39 (10.16)	24 (6.25)		
>20 years	124 (2.04)	44 (35.48)	55 (44.35)	15 (12.10)	10 (8.06)		
**Months of shift work per year**							
1–3 months	289 (4.75)	152 (52.60)	100 (34.60)	31 (10.73)	6 (2.08)	45.92	**<0.001**
10–12 months	4,757 (78.27)	1,916 (40.28)	2,016 (42.38)	551 (11.58)	274 (5.76)		
7–9 months	538 (8.85)	211 (39.22)	266 (49.44)	42 (7.81)	19 (3.53)		
4–6 months	494 (8.13)	224 (45.34)	209 (42.31)	45 (9.11)	16 (3.24)		
**Night shifts per month**							
≤ 4	1,267 (20.85)	602 (47.51)	484 (38.2)	122 (9.63)	59 (4.66)	34.89	**<0.001**
5–9	4,150 (68.28)	1,655 (39.88)	1,825 (43.98)	462 (11.13)	208 (5.01)		
≥10	661 (10.88)	246 (37.22)	282 (42.66)	85 (12.86)	48 (7.26)		
**Interval between night shifts**							
≤ 4 days	1,955 (32.17)	779 (39.85)	827 (42.30)	241 (12.33)	108 (5.52)	7.87	0.248
5–7 days	3,474 (57.16)	1,443 (41.54)	1,488 (42.83)	368 (10.59)	175 (5.04)		
≥8 days	649 (10.68)	281 (43.30)	276 (42.53)	60 (9.24)	32 (4.93)		
**Night shift staffing**							
1	1,932 (31.79)	815 (42.18)	799 (41.36)	218 (11.28)	100 (5.18)	10.58	0.306
2 (take turns)	2,212 (36.39)	916 (41.41)	953 (43.08)	220 (9.95)	123 (5.56)		
2 (take no turns)	838 (13.79)	322 (38.42)	382 (45.58)	97 (11.58)	37 (4.42)		
≥3	1,096 (18.03)	450 (41.06)	457 (41.70)	134 (12.23)	55 (5.02)		
**Psychological stress before night shifts**							
No	633 (10.41)	455 (71.88)	138 (21.8)	27 (4.27)	13 (2.05)	276.13	**<0.001**
Yes	5,445 (89.59)	2,048 (37.61)	2,453 (45.05)	642 (11.79)	302 (5.55)		
**Psychological stress during night shifts**							
No	698 (11.48)	508 (72.78)	150 (21.49)	29 (4.15)	11 (1.58)	327.28	**<0.001**
Yes	5,380 (88.52)	1,995 (37.08)	2,441 (45.37)	640 (11.9)	304 (5.65)		
**Psychological stress after night shifts**							
No	2,546 (41.89)	1452 (57.03)	879 (34.52)	154 (6.05)	61 (2.40)	498.26	**<0.001**
Yes	3,532 (58.11)	1,051 (29.76)	1,712 (48.47)	515 (14.58)	254 (7.19)		
**Using sleep medication before night shifts**							
Never/rarely	4,729 (77.81)	2,167 (45.82)	1,956 (41.36)	424 (8.97)	182 (3.85)	296.25	**<0.001**
Sometimes	1,130 (18.59)	280 (24.78)	549 (48.58)	203 (17.96)	98 (8.67)		
Often	219 (3.60)	56 (25.57)	86 (39.27)	42 (19.18)	35 (15.98)		
**Using sleep medication after night shifts**							
Never/rarely	4,930 (81.11)	2,234 (45.31)	2,046 (41.5)	453 (9.19)	197 (4.00)	288.21	**<0.001**
Sometimes	946 (15.56)	217 (22.94)	473 (50.00)	167 (17.65)	89 (9.41)		
Often	202 (3.32)	52 (25.74)	72 (35.64)	49 (24.26)	29 (14.36)		
**Physical discomfort during night shifts**							
No	1,935 (31.84)	1,151 (59.48)	637 (32.92)	117 (6.05)	30 (1.55)	429.40	**<0.001**
Yes	4,143 (68.16)	1,352 (32.63)	1,954 (47.16)	552 (13.32)	285 (6.88)		
**Busyness during night shifts**							
No	1,739 (28.61)	867 (49.86)	677 (38.93)	152 (8.74)	43 (2.47)	98.22	**<0.001**
Yes	4,339 (71.39)	1,636 (37.70)	1,914 (44.11)	517 (11.92)	272 (6.27)		
**Feeling of being refreshed after resting before night shifts**							
No	1,657 (27.26)	408 (24.62)	774 (46.71)	282 (17.02)	193 (11.65)	419.09	**<0.001**
Yes	4,421 (72.74)	2,095 (47.39)	1,817 (41.10)	387 (8.75)	122 (2.76)		
**Feeling of being refreshed after resting after night shifts**							
No	767 (12.62)	182 (23.73)	324 (42.24)	148 (19.3)	113 (14.73)	274.22	**<0.001**
Yes	5,311 (87.38)	2,321 (43.70)	2,267 (42.68)	521 (9.81)	202 (3.80)		
**Sleep quality before night shifts**							
Normal	3,996 (65.75)	1,904 (47.65)	1,645 (41.17)	326 (8.16)	121 (3.03)	314.81	**<0.001**
Poor	2,082 (34.25)	599 (28.77)	946 (45.44)	343 (16.47)	194 (9.32)		
**Sleep quality after night shifts**							
Normal	4,700 (77.33)	2,118 (45.06)	1,971 (41.94)	445 (9.47)	166 (3.53)	231.81	**<0.001**
Poor	1,378 (22.67)	385 (27.94)	620 (44.99)	224 (16.26)	149 (10.81)		
**Food intake during shift work**							
Normal	2,085 (34.30)	1,051 (50.41)	798 (38.27)	173 (8.30)	63 (3.02)	133.62	**<0.001**
More	749 (12.32)	276 (36.85)	317 (42.32)	102 (13.62)	54 (7.21)		
Less	3,244 (53.37)	1,176 (36.25)	1,476 (45.5)	394 (12.15)	198 (6.10)		
**Meal timing during shift work**							
Regular	2,302 (37.87)	1,141 (49.57)	904 (39.27)	195 (8.47)	62 (2.69)	141.66	**<0.001**
Early	1,008 (16.58)	384 (38.10)	432 (42.86)	123 (12.20)	69 (6.85)		
Delayed	2,768 (45.54)	978 (35.33)	1255 (45.34)	351 (12.68)	184 (6.65)		
**Working > 40 h/week during shift work**							
Never/rarely	1,148 (18.89)	671 (58.45)	370 (32.23)	75 (6.53)	32 (2.79)	261.40	**<0.001**
1 week/month	1,160 (19.09)	503 (43.36)	512 (44.14)	113 (9.74)	32 (2.76)		
2 weeks/month	1,069 (17.59)	401 (37.51)	494 (46.21)	134 (12.54)	40 (3.74)		
3 weeks/month	638 (10.50)	234 (36.68)	289 (45.3)	77 (12.07)	38 (5.96)		
4 weeks/month	2,063 (33.94)	694 (33.64)	926 (44.89)	270 (13.09)	173 (8.39)		
**Rest during night shifts**							
No	1,868 (30.73)	757 (40.52)	731 (39.13)	246 (13.17)	134 (7.17)	40.10	**<0.001**
Yes	4,210 (69.27)	1,746 (41.47)	1,860 (44.18)	423 (10.05)	181 (4.30)		

*SD, standard deviation; CNY, China Yuan. Statistically significant differences after application of the Bonferroni correction are shown as bold numbers (p < 0.002)*.

† *Nursing professional titles can be divided into three categories: primary, intermediate and senior*.

‡ *Other work schedules include long-term irregular shifts for a variety of reasons*.

§ *F for one-way analysis of variance (ANOVA) and χ2 for chi-square test*.

### Univariate Analysis of Demographic and Shift Work Characteristics Associated with Depression/Anxiety in Nurses

Tables 1, 2 also show the results of the univariate analysis of the depression and anxiety-related demographic characteristics in all nurses. According to the Bonferroni correction, a *p-*value <0.006 was considered as statistically significant in the above univariate analysis. We found that shift work significantly correlated with the levels of anxiety (χ^2^ = 73.54, *p* < 0.001) and depression (χ^2^ = 123.16, *p* < 0.001). Additionally, the number of years spent engaged in nursing significantly correlated with the levels of anxiety (*F* = 5.30, *p* = 0.001) and depression (*F* = 6.30, *p* < 0.001). The level of anxiety varied according to gender (χ^2^ = 16.48, *p* < 0.001), department (χ^2^ = 65.23, *p* < 0.001), and professional title (χ^2^ = 37.93, *p* < 0.001). Furthermore, the level of depression varied according to gender (χ^2^ = 25.25, *p* < 0.001), department (χ^2^ = 73.49, *p* < 0.001), and professional title (χ^2^ = 59.27, *p* < 0.001).

See Tables 3, 4 for the results of our univariate analysis of demographic and shift work characteristics related to depression and anxiety in shift nurses. According to the Bonferroni correction, a *p-*value <0.002 was considered as statistically significant in the above univariate analysis. Among the demographic variables, age was associated with anxiety level (*F* = 5.74, *p* < 0.001) and depression level (*F* = 4.61, *p* = 0.001) in shift nurses. Shift arrangement characteristics included the number of months engaged in shift work per year, the number of night shifts worked per month, working > 40 h/week during shift work, busyness during night shifts, and resting during night shifts, all of which correlated with levels of depression and anxiety. Physical and mental characteristics associated with depression and anxiety included physical fatigue/mental fatigue/physical discomfort during night shifts, psychological stress before/during/after night shifts, and the feeling of being refreshed after resting before/after night shifts. Personal habits during shift work associated with depression and anxiety levels included sleep quality before/after night shifts, using sleep medication before/after night shifts, food intake during shift work, and the timing of meals during shift work.

### Multivariable Ordinal Logistic Regression Analysis of Demographic and Shift Work Characteristics Associated with Depression and Anxiety

Before performing the multivariable ordinal logistic regression analysis, we conducted a test for multicollinearity. We substituted dummy variables for the ordinal and nominal variables, and found that no variables had a VIF value >10. Table 5 shows the results of the multivariable ordinal logistic regression analysis of demographic characteristics associated with depression (*R*^2^ = 0.345) and anxiety (*R*^2^ = 0.326) in all nurses. According to the Bonferroni correction, a *p-*value < 0.025 was considered as statistically significant in our regression analysis. The fully adjusted model indicated that shift work correlated with higher levels of depression and anxiety among all nurses (OR = 1.54, 95% CI: 1.40–1.69; and OR = 1.36, 95% CI: 1.24–1.50, respectively) when other demographic characteristics were held constant.

**Table 5:** Multivariable ordinal logistic regression analysis of demographic characteristics associated with depression and anxiety in all nurses (*N* = 9,181).

**Variables**	**Depression**		**Anxiety**	
	**OR (95% CI)**	***p* value**	**OR (95% CI)**	***p* value**
Age (for every additional year)	1.00 (0.99, 1.02)	0.673	1.00 (0.99, 1.02)	0.442
Years of working (for every additional year)	1.00 (0.99, 1.01)	0.686	0.99 (0.98, 1.00)	0.232
**Gender (ref: Male)**				
Female	0.74 (0.61, 0.89)	**0.001**	0.83 (0.69, 1.01)	0.056
**Department (ref: Internal medicine)**				
Surgery	0.94 (0.84, 1.05)	0.251	0.92 (0.83, 1.03)	0.148
Emergency	0.99 (0.83, 1.19)	0.941	0.98 (0.82, 1.18)	0.856
Gynecology and obstetrics	0.78 (0.66, 0.91)	**0.002**	0.77 (0.65, 0.90)	**0.001**
Pediatrics	0.89 (0.76, 1.04)	0.135	0.92 (0.79, 1.08)	0.316
Operating room	0.79 (0.67, 0.94)	**0.007**	0.85 (0.72, 1.01)	0.061
Intensive care unit	1.07 (0.90, 1.27)	0.428	1.07 (0.90, 1.27)	0.466
Outpatient	0.98 (0.78, 1.22)	0.842	0.77 (0.62, 0.97)	0.026
Others	0.81 (0.70, 0.92)	**0.002**	0.81 (0.71, 0.93)	**0.003**
**Professional title^†^ (ref: Primary)**				
Intermediate	1.22 (1.10, 1.36)	**<0.001**	1.24 (1.11, 1.38)	**<0.001**
Senior	0.81 (0.63, 1.04)	0.096	0.90 (0.70, 1.15)	0.396
**Shift work (ref: No)**				
Yes	1.54 (1.40, 1.69)	**<0.001**	1.36 (1.24, 1.50)	**<0.001**

*OR, odds ratio; CI, confidence interval; ref, reference group. Statistically significant differences after application of the Bonferroni correction are shown as bold numbers (p < 0.025)*.

† *Nursing professional titles can be divided into three categories: primary, intermediate and senior*.

We next sought to determine the specific characteristics of shift work that affect depression (*R*^2^ = 0.412) and anxiety (*R*^2^ = 0.392) levels (Table 6). We found that in shift nurses, overtime work correlated with higher depression and anxiety levels. The ORs of depression and anxiety continuously increased with the amount of overtime work (i.e., from 1 week to 4 weeks per month), indicating that more overtime work increases the chance of depression and anxiety. Busyness during night shifts (OR = 1.44, 95% CI: 1.01–1.28) and overeating during shift work correlated with higher level of depression in shift nurses (OR = 1.25, 95% CI: 1.04–1.50).

**Table 6:** Multivariable ordinal logistic regression analysis of demographic and shift work characteristics associated with depression and anxiety in shift nurses (*N* = 6,078).

**Variables**	**Depression**		**Anxiety**	
	**OR (95% CI)**	***p* value**	**OR (95% CI)**	***p* value**
Age (for every additional year)	0.99 (0.98, 1.00)	0.139	0.99 (0.98, 1.00)	0.197
**Months of shift work per year (ref: 1–3 months)**				
10–12 months	1.24 (0.97, 1.58)	0.088	1.22 (0.95, 1.57)	0.122
7–9 months	1.06 (0.79, 1.42)	0.699	1.24 (0.93, 1.67)	0.149
4–6 months	1.15 (0.85, 1.54)	0.364	1.13 (0.84, 1.54)	0.412
**Night shifts per month (ref: ≤4)**				
5–9	0.95 (0.84, 1.09)	0.476	0.95 (0.83, 1.08)	0.406
≥10	1.06 (0.88, 1.28)	0.562	0.91 (0.75, 1.11)	0.349
**Psychological stress before night shifts (ref: No)**				
Yes	1.09 (0.83, 1.42)	0.535	1.14 (0.87, 1.50)	0.343
**Psychological stress during night shifts (ref: No)**				
Yes	1.30 (1.01, 1.69)	0.046	1.51 (1.16, 1.98)	**0.002**
**Psychological stress after night shifts (ref: No)**				
Yes	1.51 (1.35, 1.69)	**<0.001**	1.73 (1.54, 1.94)	**<0.001**
**Feeling of being refreshed after resting before night shifts (ref: No)**				
Yes	0.72 (0.64, 0.81)	**<0.001**	0.69 (0.61, 0.78)	**<0.001**
**Feeling of being refreshed after resting after night shifts (ref: No)**				
Yes	0.71 (0.61, 0.83)	**<0.001**	0.75 (0.64, 0.88)	**<0.001**
**Using sleep medication before night shifts (ref: Never/rarely)**				
Sometimes	1.23 (1.04, 1.45)	**0.015**	1.23 (1.04, 1.46)	**0.017**
Often	1.54 (1.09, 2.16)	**0.014**	1.59 (1.13, 2.22)	**0.007**
**Using sleep medication after night shifts (ref: Never/rarely)**				
Sometimes	1.42 (1.19, 1.70)	**<0.001**	1.28 (1.07, 1.53)	**0.007**
Often	1.67 (1.17, 2.38)	**0.005**	1.35 (0.95, 1.93)	0.095
**Physical discomfort during night shifts (ref: No)**				
Yes	1.62 (1.43, 1.83)	**<0.001**	1.46 (1.29, 1.65)	**<0.001**
**Busyness during night shifts (ref: No)**				
Yes	1.14 (1.01, 1.28)	**0.030**	1.06 (0.95, 1.20)	0.300
**Sleep quality before night shifts (ref: Normal)**				
Poor	1.19 (1.06, 1.33)	**0.004**	1.19 (1.06, 1.34)	**0.004**
**Sleep quality after night shifts (ref: Normal)**				
Poor	1.29 (1.14, 1.47)	**<0.001**	1.18 (1.04, 1.34)	**0.012**
**Food intake during shift work (ref: Normal)**				
More	1.25 (1.04, 1.50)	**0.019**	1.10 (0.91, 1.33)	0.320
Less	1.12 (0.98, 1.29)	0.096	1.03 (0.90, 1.19)	0.644
**Meal timing during shift work (ref: Regular)**				
Early	1.02 (0.86, 1.20)	0.820	1.12 (0.95, 1.33)	0.181
Delayed	1.13 (0.98, 1.29)	0.084	1.13 (0.98, 1.29)	0.089
**Working > 40 h/week during shift work (ref: Never/rarely)**				
1 week/month	1.29 (1.09, 1.52)	**0.003**	1.27 (1.07, 1.51)	**0.007**
2 weeks/month	1.41 (1.18, 1.67)	**<0.001**	1.47 (1.23, 1.75)	**<0.001**
3 weeks/month	1.41 (1.16, 1.72)	**0.001**	1.51 (1.23, 1.84)	**<0.001**
4 weeks/month	1.60 (1.37, 1.87)	**<0.001**	1.66 (1.42, 1.94)	**<0.001**
**Fatigue scores (for every additional point)**				
Physical fatigue scores	1.20 (1.18, 1.23)	**<0.001**	1.19 (1.17, 1.22)	**<0.001**
Mental fatigue scores	1.35 (1.30, 1.40)	**<0.001**	1.26 (1.21, 1.31)	**<0.001**
**Rest during night shifts (ref: No)**				
Yes	1.03 (0.92, 1.15)	0.632	0.97 (0.87, 1.09)	0.632

*OR, odds ratio; CI, confidence interval; ref, reference group. Statistically significant differences after application of the Bonferroni correction are shown as bold numbers (p < 0.025)*.

Shift nurses often plan rest periods before and after night shifts. Therefore, we explored the effect of energy recovery after rest on depression and anxiety in shift nurses. Our results showed that the feeling of being refreshed after resting before a night shift significantly correlated with lower levels of depression and anxiety in shift nurses (OR = 0.72, 95% CI: 0.64–0.81; and OR = 0.69, 95% CI: 0.61–0.78, respectively). Similarly, the feeling of being refreshed after resting after a night shift was associated with reduced depression and anxiety in shift nurses (OR = 0.71, 95% CI: 0.61–0.83; and OR = 0.75, 95% CI: 0.64–0.88, respectively). We investigated whether the quality of sleep before and after night shifts affected depression and anxiety levels in shift nurses. Our results showed that poor sleep quality before or after night shifts was associated with greater levels of anxiety (OR = 1.19, 95% CI: 1.06–1.34; and OR = 1.18, 95% CI: 1.04–1.34, respectively) and depression (OR = 1.19, 95% CI: 1.06–1.33; and OR = 1.29, 95% CI: 1.44–1.47, respectively) in shift nurses. Moreover, both pre- and post-shift use of sleep medication significantly correlated with greater levels of depression and anxiety.

We next examined the effects of shift-related physical and psychological factors on mental health. We found that psychological stress related to night shifts negatively impacted depression and anxiety in shift nurses according to the time period. For example, psychological stress during night shifts led to higher anxiety levels (OR = 1.51, 95% CI: 1.16–1.98), whereas psychological stress after night shifts led to higher levels of both anxiety (OR = 1.73, 95% CI: 1.54–1.94) and depression (OR = 1.51, 95% CI: 1.35–1.69). Furthermore, physical discomfort during night shifts was a risk factor for increased levels of depression and anxiety (OR = 1.62, 95% CI: 1.43–1.83; and OR = 1.46, 95% CI: 1.29–1.65, respectively). Finally, physical fatigue correlated with greater levels of depression and anxiety (OR = 1.20, 95% CI: 1.18–1.23; and OR = 1.19, 95% CI: 1.17–1.22, respectively), as was the case with psychological fatigue (OR = 1.26, 95% CI: 1.21–1.31; and OR = 1.26, 95% CI: 1.21–1.31, respectively). The ORs of depression and anxiety for psychological fatigue were greater than those for physical fatigue.

## Discussion

### Prevalence of Depression and Anxiety Among Nurses

In this study, 55.85% of all nurses surveyed, and 58.82% of shift work nurses surveyed reported varying levels of anxiety. This prevalence was higher than that reported by health care workers in a previous Chinese study (44.6%) (36) and roughly the same as that in a Spanish study (58.6%) (37). In this study, 14.66–16.19% of nurses showed moderate to severe anxiety, which was higher than that in Japanese nurses (11.10%) (38). In addition, 58.39% of all nurses surveyed, and 62.08% of shift work nurses surveyed had varying degrees of depression, which was higher than the prevalence reported by Lai et al. in China (50.4%) (36), that reported by Awano et al. in Japan (34.90%) (38), and that reported by Luceño-Moreno et al. in Spain (46.0%) (37). The prevalence of depression among nurses in the Korean Nurses' Health Cohort was 64.80%, which was roughly the same as that in our study (25). These differences in the prevalence of depression and anxiety may be related to varying regional and social backgrounds among study populations. In the context of infection prevention and control during the COVID-19 pandemic, nurses in China had a heavier workload and increased shift work pressure, which is likely to have affected their mental health (39).

### Effect of Shift Work Characteristics on Depression and Anxiety

A previous study found that self-reported levels of depression and anxiety were higher in shift workers than in non-shift workers (7). In the present study, shift nurses were 1.54 and 1.36 times more likely to have greater levels of depression and anxiety, respectively, compared with non-shift nurses. A meta-analysis of longitudinal data showed that shift work enhanced the overall risk of adverse mental health outcomes (e.g., depression and anxiety) by 28% among 28,431 participants (22). Lee et al. also found that Korean nurses who engaged in shift work were 1.5 times more likely to experience more severe depressive symptoms in comparison with non-shift workers (25). A previous population-based study surveying 277,168 workers in the UK Biobank indicated that shift workers were more likely to consult a physician for treatment of feelings of depression, anxiety, emotional instability, or neuroticism (3).

Lee et al. recently conducted a meta-analysis of observational epidemiological studies. They found that shift work at night was correlated with a 40% increase in the risk of depression, which persisted in subgroup analyses by shift duration, sex, and occupation (13). Similarly, in a longitudinal study of UK households, Weston et al. discovered that shift work significantly correlated with depressive symptoms regardless of occupation, age, or sex (24). The above study was consistent with our findings in that we found no statistically significant correlation between gender, age, shift duration, or depressive symptoms in nurses. However, our finding may be because female nurses accounted for a large proportion of the shift nurses in this study, i.e., 93.37%. Therefore, the gender imbalance should be considered for our data interpretation. Future studies should seek to increase the sample size of male nurses to obtain more representative information regarding mental health outcomes in male nurses (40).

Most studies that have compared different patterns of shift work in terms of negative psychological outcomes have been highly dependent on general demographic factors. Thus, further investigation is needed regarding differences in shift work characteristics, personal factors, and the environmental characteristics of shift work (41). In this study, we sought to address gaps in previous work by exploring individual factors and characteristics of the shift work environment. We found that the number of night shifts worked, the lengths of shifts, the work schedule and the shift rotation direction did not influence depression and anxiety among shift nurses. These findings are supported by Øyane et al. (18), Berthelsen et al. (42), and Lin et al. (16). Some possible reasons for the above results were as follows. First, we may have not collected the most important variables among shift-related variables, such as night shift handover time, shift adaptability, circadian rhythm changes during shifts, etc., which need to be supplemented in future studies. Second, circadian rhythm changes during shifts may fully mediate the relationship between the above variables and anxiety/depression, leading to the possibility that these variables may not directly affect mental health (6, 7).

Berthelsen et al. (42) and Natvik et al. (43) found that the number of h worked per week did not correlate with an increased incidence of anxiety or depression. However, other studies focused on extended working h (overtime) have found a positive linear correlation between the additional h worked as a result of overtime and anxiety or depression (8, 44). Our findings are consistent with the above studies, in which we found a positive dose-response relationship between the number of overtime weeks per month and anxiety or depression. Furthermore, we found the proportion of nurses who worked at least some overtime each month to be as high as 81.11%. These results suggest that overtime work may be not conducive to the maintenance of physical and mental health in shift nurses. Nursing managers should seek to understand the reasons why nurses engage in overtime work as well as the negative impacts on mental and physical health, and thus make changes to the workplace to limit this type of work practice.

Altered sleep patterns caused by shift work have been linked with irritability, depressed mood, anxiety, and nervousness (45, 46). A prospective study of shift workers found that changes in mental health, including symptoms of depression and anxiety, were modulated by sleep after engaging in shift work for 1 year (47). Based on the above findings, we explored the effects of sleep and energy recovery at different times relative to shift work on depression and anxiety in nurses. Shift nurses often plan sleep or rest periods before and after night shifts to ensure that they have an adequate energy level for working and are restored after the experience of shift work. Our present data indicate that shift nurses who self-reported poor sleep quality before or after night shifts may have an increased risk of anxiety or depression. At the same time, we found that nurses who self-reported that their energy levels returned to normal after pre- or post-night shift sleep may be at reduced risk of anxiety or depression. Our results are supported by a study by Booker et al. who found that disordered sleep as a result of shift work led to more severe symptoms of depression and anxiety (48). It is possible that poor sleep quality and low energy levels lead to reduced enthusiasm regarding shift work. Such negative emotions could accumulate, leading to increased symptoms of depression and anxiety, and ultimately decreased work efficiency (49). Our data indicate that nurses who used sleep medications during sleep before or after night shifts were more likely to report higher levels of anxiety or depression. However, we know that nurses with higher anxiety/depression may be more likely to take sleep medications. Given the cross-sectional nature of this study, the direction of potential causality may not be known. Therefore, longitudinal data may need to be collected to further explore the relationship. In addition, nursing managers may benefit from evaluating the sleep quality and sleep drug use of shift nurses, and aim to provide a good sleep environment and sleep hygiene advice for night nurses. These measures could reduce the negative effects of sleep problems on levels of depression and anxiety in shift nurses.

Our results suggest that night shift busyness may be a factor for increased risk of depression and anxiety severity among nurses, and that busy night shifts are a possible cause of fatigue (50). Previous studies have shown that shift-related fatigue is significantly connected with depression and anxiety in nurses (51, 52). In this study, we discriminated mental fatigue from physical fatigue, and found that both factors may increase the risk of depression and anxiety in shift nurses. Therefore, from the perspective of nursing management, reducing the work burden of shift nurses and helping them lower the degree of physical and mental fatigue will likely reduce the severity of symptoms of depression and anxiety. After exploring the relationship between psychological stress at different time periods of shifts and the mental health of shift nurses, we found that psychological stress before night shifts, psychological stress during night shifts, and psychological stress after night shifts may be all risk factors for depression and anxiety in shift nurses. Previous studies have shown a significantly positive correlation between stress levels in shift nurses and symptoms of depression and anxiety (37, 52). Further studies are needed to determine the causes of psychological stress in shift nurses during night shifts to reduce the impact of this stress on their mental health (e.g., depression and anxiety).

### Limitations and Strengths

First, although this was a multi-center study, it had a cross-sectional design. Thus, we were able to conduct correlational analyses but not causal inferences. Further studies with a longitudinal design may support our results. Second, the data in this study were subjective, which may have led to partial recall bias. Future studies with objective measurements are needed to strengthen our findings. Despite the above shortcomings, we have contributed to the field by exploring the detailed relationships between shift-related variables at different time periods and depression and anxiety in shift nurses. In addition to the frequency and direction of rotation of shift schedules, we also examined psychological stress, dietary changes, and fatigue levels during night shifts.

## Conclusion

The characteristics of shift work such as overtime work and busy night shift work may be not conducive to the mental health of shift nurses. Moreover, psychological stress and fatigue during night shifts appear to enhance symptoms of depression and anxiety. Physical discomfort and fatigue during night shifts may have negative impacts on mental health. Efforts to decrease depression and anxiety in shift nurses may be carried out from the perspective of reducing the workload, reducing sources of stress during night shifts, and guiding nurses to relax and relieve fatigue.

## Data Availability Statement

The datasets presented in this article are not readily available because the data from the Nurses' Health Cohort Study of Shandong needs time for data clearing and establishment of guidelines. We are planning on opening this data to the public in the future. Requests to access the datasets should be directed to YC, caoyj@sdu.edu.cn.

## Ethics Statement

The studies involving human participants were reviewed and approved by the Ethics Committee of Scientific Research of Shandong University Qilu Hospital. The patients/participants provided their written informed consent to participate in this study.

## Author Contributions

YL and YW: methodology, formal analysis, data curation, software, writing-original draft, and visualization. RL and XL: writing-review and editing and project administration. XG, LL, and JL: investigation. YC: conceptualization, resources, supervision, project administration, and funding acquisition. All authors contributed to the article and approved the submitted version.

## Funding

This study was funded by the National Key Research and Development Program of China (Grant Number 2020YFC2003500). The funders had no role in study design, data collection and analysis, decision to publish, or preparation of the manuscript.

## Conflict of Interest

The authors declare that the research was conducted in the absence of any commercial or financial relationships that could be construed as a potential conflict of interest.

## Publisher's Note

All claims expressed in this article are solely those of the authors and do not necessarily represent those of their affiliated organizations, or those of the publisher, the editors and the reviewers. Any product that may be evaluated in this article, or claim that may be made by its manufacturer, is not guaranteed or endorsed by the publisher.
